# Development and validation of Child-Friendly School Environment Questionnaire from Chinese culture

**DOI:** 10.3389/fpsyg.2023.1288085

**Published:** 2023-11-27

**Authors:** Erping Xiao, Mengju Sun, Kexin Lv, Xinyi Zhu, Wenbin Jia

**Affiliations:** ^1^Zhejiang Philosophy and Social Science Laboratory for Research in Early Development and Childcare, Hangzhou Normal University, Hangzhou, China; ^2^Jing Hengyi School of Education, Hangzhou Normal University, Hangzhou, China

**Keywords:** child-friendly environment, school environment, evaluation tool, Chinese, Child-Friendly School Environment Questionnaire

## Abstract

In the context of building Child-Friendly Cities in China, child-friendly school environments are considered as having a profound impact on children’s development and growth. This study presents the development and validation of the Child-Friendly School Environment Questionnaire for assessing a child-friendly school environment. Utilizing open-ended questions and interviews, an initial questionnaire on the child-friendly school environment was compiled. An exploratory factor analysis of the preliminary test results with 696 primary school children in grades three to six was conducted to refine the questionnaire into a formal 19-item questionnaire. Subsequently, a confirmatory factor analysis was performed to analyze the evaluation results of 807 primary school children in grades three to six. The results indicated that a child-friendly school environment is a multi-dimensional construct encompassing Environment Friendly, Teaching Friendly, Peer Friendly, and Children Participation, with good reliability and validity. The promising outcomes of this study suggest that the Child-Friendly School Environment Questionnaire can be widely used as a powerful evaluation tool for the child-friendly school education practice in the future.

## 1 Introduction

Environmental quality is closely related to children’s development. A child-friendly environment is a community-driven initiative that arises from local structures that extend beyond the individual level (Horelli, 1998). It refers to the provision of settings and environmental structures that facilitate the support of individual children and groups with an interest in children’s issues, enabling them to effectively develop and execute their own goals and projects (Horelli, 2007). Child-friendly environments encompass various settings, including child-friendly cities, communities, hospitals, and spaces. These spaces can include integrated public spaces, urban environments, play spaces, routes, planning, tourism environments, and high-density environments or communities (Hanaba, 2018; Bhandari, 2020). Child-friendly environments play a crucial role in creating secure public spaces and fostering social cohesion and a sense of community (Brown et al., 2019; Nasrabadi et al., 2021).

Studies on the dimensions of child-friendly environments have revealed variations across different countries and age groups. Based on data collected from 12-year-olds in Finland and Italy, Haikkola et al. (2007) formulated a theoretical framework consisting of 10 dimensions of a child-friendly environment. These dimensions encompass various aspects such as housing and dwelling, basic services, participation, safety and security, family, kin, peers and community, urban and environmental qualities, provision and distribution of resources, ecology, sense of belonging and continuity, and good governance (Horelli, 2007). Three dimensions were specifically applied to children’s responses: safety and security, urban and environmental qualities, and basic services (Nordstrom, 2010). A child-friendly environment is a comprehensive framework that facilitates children’s holistic development, including individual growth and the development of related vital groups for the child. This concept is characterized by its complexity, multidimensionality, and multilevel nature. It is imperative to establish a secure environment for every child, encompassing physical, emotional, and psychological safety (Bhandari, 2020).

For children, apart from the family environment, the school environment also significantly impacts on an individual’s development. The school environment plays a crucial role in ensuring the safety and well-being of students, encompassing various aspect such as the physical, academic, and social environment (Greenberg et al., 2003; Lawrence and Vimala, 2012). The school environment significantly impacts students’ moral and cultural literacy through various learning activities, which in turn can influence their overall well-being (Maryani et al., 2019). As widely acknowledged, the main aim of schooling, particularly in the context of primary education, is not merely to transmit knowledge but rather to cultivate an authentic enthusiasm for learning in students, independent of any external motivations (Barrow and Woods, 2006). Thus, creating an environment where students enjoy learning for their interests is the primary purpose of school.

Previous studies indicated that the learning environment plays a significant role in influencing students’ learning outcomes. The promotion of students’ learning initiative and enhancement of their academic performance have been observed in previous studies (Allen and Fraser, 2007; Aldridge and Fraser, 2011; Aldridge et al., 2012). Appropriate environmental stimuli play a crucial role in shaping the cognitive development of children by facilitating their interaction with the environment and fostering their acceptance of environmental influences (Ambarsari and Harun, 2019). The factors that that influence the school environment, including the setting of school ecology, the setting of humanistic atmosphere, and the integration of school culture in environmental design, are all able to create a unique campus culture (Melin et al., 2022). School tremendously impacts an individual’s life. However, it is important to note that the impact of school experiences on children can vary depending on the school environment (Fitriani et al., 2021). Therefore, the establishment of a child-friendly school environment that is conducive to the needs and well-being of children is imperative in order to facilitate sustainable development in education.

The child-friendly school is considered a significant initiative in promoting sustainable education development. This approach aims to create a hygienic and conductive learning environment while prioritizing children welfare in the school (Ambarsari and Harun, 2019). According to the United Nations Educational, Scientific, and Cultural Organization (UNESCO), a fundamental aim of sustainable development is to ensure healthy life and enhance individuals’ overall welfare. Sustainable development offers a potential approach to addressing environmental issues, exploring alternatives solutions, and assuming responsibility for the outcomes of actions and decisions (Summers et al., 2005).

UNICEF has developed a comprehensive framework aimed at establishing child-friendly educational systems and schools based on children’s rights. Child-friendly schools are distinguished by their commitment to inclusivity, promoting the well-being and safety of students, fostering practical learning experiences, and actively involving children, families, and communities in the holistic development of children (Osher et al., 2009). UNICEF advocates for the creation of child-friendly school models, which aim to promote the quality of education by creating a positive and supportive school environment that prioritizes health, safety, and protection (Ghavifekr and Pillai, 2016). Child-friendly schools significantly impact children by introducing concepts of respect, equality, and rights to elementary school students. These schools aim to ensure the full participation of all individuals involved, thereby promoting children’s right to receive a quality education. Additionally, the involvement of various stakeholders, such as parents, families, teachers, principals, educational administrators, civil society organizations, and local and national governments, is essential (Fitriani and Istaryatiningtias, 2020).

Child-friendly school has been widely implemented in numerous countries following the proposal by UNICEF in 2009. A case study was conducted in Pakistan schools to explore teachers’ role in developing child-friendly environment in Early Childhood Education classrooms, which revealed that institutional support and monitoring teachers’ personal propensity to learning for improving students’ learning (Murtaza, 2011). In Nepal’s disadvantaged schools, emphasis was placed on child-friendly environments (Khanal, 2021). In recent years, the Chinese government has placed significant emphasis on advancing the construction of child-friendly cities and has issued “Guidance on Promoting the Construction of Child-friendly Cities” across the nation in 2021. As a result, all levels of government have implemented pertinent local policies. Shenzhen took the initiative in China by becoming the first city to embark on the development of a child-friendly city. Additionally, it introduced the first local standard for the construction of child-friendly cities in China. In child-friendly city development, the construction of child-friendly schools is also a key focus. “Guidelines for the Construction of Child-friendly Schools (Primary and Secondary Schools) in Shenzhen (Revised Version)” were jointly issued by the Shenzhen Municipal Working Committee on Women and Children Committee Member, Shenzhen Education Bureau, and Bureau of Public Works of Shenzhen Municipality. These guidelines aim to promote the purpose of school operation, advocate the concept of “green, ecological and sustainable development,” and by means of the design of school space and facilities, as well as environmental renovation. The overarching objective of the school is to be “space friendly, facility friendly and service friendly” by implementing design principles of “safety, green, fun, humanity and barrier-free.” This document serves as the inaugural official publication on establishing child-friendly schools in China, playing a significant guiding role.

A considerable number of scholars in China have advocated for the establishment of child-friendly schools, adopting a perspective that emphasizes the “one-meter height” approach. These scholars have conducted related theoretical and practical research in this field. A child-friendly school environment should be constructed based on children’s perspective, with the “child equality” principle as a foundation to guarantee equitable and high-quality education. Additionally, “child health” should be prioritized to establish a safety net, while “children’s participation” should be emphasized to create a platform for independent growth. Lastly, the focus should be on promoting “children’s enjoyment of learning” as a critical element in facilitating effective teaching (Lu, 2019). In creating child-friendly schools, it is imperative to prioritize the inclusion of children’s perspectives and open channels for their active involvement in school management (Zhang, 2022). Meanwhile, it is also necessary to comprehend four crucial aspects: the evolution of concepts and building consensus; formulating programs and implementing actions; promoting and guiding joint participation; and piloting and demonstrating to promote comprehensively (Zhou, 2018). Children’s education should be approached from a child-centered perspective, taking into consideration the principles of their physical and mental development, as well as the need for intensive cultivation. Creating a child-perspective-friendly school environment is an essential step to ensuring children’s physical and mental health and promoting their healthy growth and comprehensive and individual development (Ding, 2021). A child-friendly school is characterized by its commitment to providing a secure and nurturing environment for students. This entails ensuring that the school has enough qualified staff members and well-trained teachers with access to appropriate resources and conducive learning conditions. This emphasis on creating a safe and supportive educational setting is recognized as a top priority (UNICEF, 2009).

In conclusion, previous studies have mainly focused on qualitative research and practical research, with less attention given to the development of evaluation tools, and they have not tested the reliability and validity of the evaluation tools from the perspective of psychometrics. The lack of a suitable quantitative evaluation tool remains a limitation in constructing a child-friendly school environment. It is necessary to examine the child-friendly school environment from the children’s perspective. Although the framework of child-friendly schools is mentioned in formal official documents, it is mainly proposed from a theoretical perspective and is not validated by empirical data and statistical methods. Examining previous research conducted in various countries and cultures will offer valuable insights for investigating child-friendly school environments within Chinese culture.

Considering the information mentioned above, the current study has two main purposes. The primary purpose of this study is to develop and validate an evaluation tool for assessing the quality of a child-friendly school environment. Using this tool, teachers in the schools could recognize the status of environmental child-friendliness, which can subsequently inform adjustments to school management and infrastructure development. In this sense, the evaluation tool serves the purpose of assessing the child-friendliness of the school environment from the perspective of the children. Additionally, it offers recommendations to school administrators for improvement. The second purpose is to ascertain the potential dimensions of a child-friendly school environment within Chinese culture. In this sense, comprehending the interplay between these dimensions can also aid in implementing interventions within the educational setting to improve children’s overall development.

## 2 Materials and methods

### 2.1 Design

The current study aimed to develop and validate the Child-Friendly School Environment Questionnaire (CFSEQ). The questionnaire was developed in three stages: item development, scale development, and scale evaluation (Boateng et al., 2018). In the first stage, an item pool was developed based on a literature review, open-ended questions and semi-structured interviews. In the second stage, an Exploratory Factor Analysis (EFA) was conducted using data from the preliminary test to ensure that each item met the statistical indicators and to formalize the questionnaire. Finally, a Confirmatory Factor Analysis (CFA) was conducted to verify the validity of the questionnaire.

### 2.2 Samples

A cluster sampling method was employed to enroll students in primary schools. Due to the limited independent reading and writing abilities of first and second graders, these participants were excluded from the present study. A total of 1,697 primary school students ranging from Grade 3 to Grade 6 participated in the current study. The participants in this study were exclusively chosen from public primary schools in Zhejiang Province, located in eastern China. The Chinese government funds public schools, ensuring that all students between the ages of 6 and 15 are mandated to receive compulsory education. Additionally, most families of the participants possess an economic status that exceeds the national average.

Three samples participated in different stages of the study. A sample of 194 children participated in the item development stage by means of open-ended questions and semi-structured interviews. Another 696 children were initially tested using the CFSEQ. The third sample (*n* = 807) was tested to verify the validity of the CFSEQ. Table 1 presents the demographic information of all participants involved in the study.

**Table 1:** Demographic information of participants for each sample.

	Sample 1	Sample 2	Sample 3
Sample size (*N*)	194	696	807
Age (*M* ± *SD*)	10.69 ± 1.10	10.60 ± 1.11	10.62 ± 1.09
Gender (*n, %*)			
Boys	100 (51.5%)	353 (50.7%)	420 (52.0%)
Girls	94 (48.5%)	343 (49.3%)	387 (48.0%)
Grade (*n, %*)			
3	45 (23.2%)	175 (25.1%)	191 (23.7%)
4	52 (26.8%)	211 (30.3%)	258 (32.0%)
5	48 (24.7%)	166 (23.9%)	192 (23.8%)
6	49 (25.3%)	144 (20.7%)	166 (20.5%)

The study was approved by the Scientific Research Ethics Committee of Hangzhou Normal University (approval number: 2022019). The legal guardians of the participants were informed about the content and procedures of the study, and provided written consent for their children to participate. Additionally, the minors themselves also provided oral consent before participating in the study.

### 2.3 Procedure

#### 2.3.1 Open-ended questions and semi-structured interviews

Initially, a literature review was conducted to summarize the concept of a child-friendly school environment (see the Introduction section). Subsequently, open-ended questions and semi-structured interviews were administered to gather additional information from the children’s perspective as a supplement. The open-ended questions were distributed by the classroom teachers, who encouraged students to provide as many details as possible based on prompts aimed at exploring their perception of a child-friendly school environment. To supplement the open-ended questions, some students (*n* = 15) were interviewed individually.

The open-ended questions and semi-structured interviews focused primarily on the following information: (1) the children’s understanding on the term “child-friendly”; (2) characteristics of child-friendly and non-child-friendly behavior; (3) characteristics of child-friendly and non-child-friendly environment; (4) approaches for implementing a child-friendly environment.

Based on previous research literature, a theoretical framework of a child-friendly school environment was developed. The information obtained from the open-ended questions and interviews was coded and integrated into the theoretical framework. The compiled framework was then repeatedly discussed and the necessity and importance of each item was validated by an expert panel (*n* = 4). The expert panel consisted of one specialist in the field of child education, one researcher in the field of child psychologists, and two primary school principals. Finally, the initial draft of the CFSEQ comprised 36 items.

#### 2.3.2 Preliminary test

A preliminary test was conducted to enhance the content validity of the questionnaire. The test involved distributing a paper version of the questionnaire to students in classrooms and asking them to complete it independently. The initial CFSEQ was presented in the form of a self-reported questionnaire using a five-point Likert scale of agreement (1 = disagree strongly; 2 = disagree a little; 3 = neither agree nor disagree; 4 = agree a little; 5 = agree strongly).

The main purpose of this preliminary test was to perform psychometric analysis of the items. Through statistical analysis, items that did not meet the requirements for internal consistency and validity index were removed, resulting in a 19-item formal questionnaire.

#### 2.3.3 Formal test

The newly developed 19-item questionnaire was used in the formal test to verify its validity. Reliability and validity were analyzed, and the structural model of the CFSEQ was obtained through Confirmatory Factor Analysis.

## 3 Results

### 3.1 Analysis of open-ended questions and interviews

The information from the texts of the open-ended questions and the interviews was recorded and transcribed. NVivo (Version 11.0) was used for coding and sorting the transcribed data to perform a qualitative analysis. The main category was extracted by merging the initial categories with the same concept connotation. Additionally, the initial category was the merging of the initial concepts with the same connotation (Wang and Fu, 2022). The results of keyword extraction and categorization were displayed in Table 2.

**Table 2:** Coding system of the open-ended questions and interviews.

Dimension	Main category	Sub-category	Concept
Environment Friendly	Social environment	Social concern	Good social environment.
	School environment	Physical environment	Playgrounds, gym, library, and medical office.
		Ecological environment	Various kinds of food, plants, and animals.
		Space environment	Safe, clean, free, quiet, and happy.
	Classroom environment	Physical environment	Intelligent devices and book corner.
		Space environment	Clean, happy, and free.
Relationship friendly	Teacher-student relationship	Democratic and equal	No punishment from teachers, students’ opinions are valued.
		Patience	More patient and caring to students.
		Mutual respect	Teachers and students respect each other.
	Peer relationship	Friendly behavior	No fight or swear, no bulling.
		Friendly language	Peers help each other, polite language.

Regarding the understanding of the term “child-friendly,” children perceive it as receiving special care, friendly behaviors, and every possible consideration and respect. In terms of the characteristics of child-friendly and non-child-friendly behavior, choosing freely and actively helping and caring for others are considered friendly, while bullying, conflict, physical or psychological punishment are considered unfriendly. For the characteristics of child-friendly and non-child-friendly environments, delicious food, spacious playgrounds, and enough free time and space are mentioned as friendly, while crowded spaces, disregard by others, and lack of principal’s suggestion boxes are mentioned as unfriendly. The implementation approach for a child-friendly environment involves a school where children can care for pets and grow plants, and where the school environment is clean and orderly.

### 3.2 Analysis of preliminary test

#### 3.2.1 Discrimination test

The discrimination test for the initial questionnaire was carried out by first calculating the total score and arranging them in order of the sum, with the top 27% of subjects forming the high-scoring group and the bottom 27% forming the low-scoring group. An independent samples *t*-test was conducted on the mean scores of these two groups to eliminate items with non-significant differences (Wang and Li, 2020). It was then concluded that all items were significant (*ps* < 0.05).

#### 3.2.2 Exploratory factor analysis (EFA)

Exploratory factor analysis is based on the data collected from the original 36-item questionnaire. The criteria for determining whether the data is suitable for exploratory factor analysis mainly include sample size, Bartlett’s test of sphericity, and Kaiser-Meyer-Olkin (KMO) index (Comrey and Lee, 1992; Nunnally and Bernstein, 1994; DeVellis, 2016). In the current study, the sample size of 696 participants is considered appropriate for exploratory factor analysis, as the recommended minimum sample size is 300. The results of Bartlett’s sphericity test of sphericity, *χ^2^*(171) = 6242.32, *p* < 0.001, meet the requirements for exploratory factor analysis. The KMO index in this study is 0.93, indicating that the data is suitable for exploratory factor analysis with adequate fit (above 0.60).

To determine the number of factors to retain, principle component analysis (PCA) as well as the maximum variance rotation method were performed (Tabachnick and Fidell, 2013). Multiple criteria were used for determining the number of factors and item removal. Items which have factor loadings below 0.40, cross-loadings on more than two factors with loading values above 0.32, and have a communality coefficient below 0.30 would be removed (Comrey and Lee, 1992; Davidson et al., 2023). An exploratory factor analysis and internal reliability analysis were run repeatedly each time an item was removed, ensuring that deletion would minimize the impact on the factor structure or internal consistency (Costello and Osborne, 2005; Worthington and Whittaker, 2006; Tabachnick and Fidell, 2013). Based on the above criteria, 19 items were retained. The Cronbach’s alpha for the remaining 19 items was 0.87, indicating good internal consistency of the items (Nunnally and Bernstein, 1994; Hinkin, 1998).

After item removal, the four-factor model maintained the most interpretable structure and clear factor loadings. Additionally, this model was most conceptually relevant to the multi-dimensional model of a Child-friendly School Environment. Therefore, factors that could not be interpreted meaningfully were not retained. Based on the content of the items, the four factors were named Environment Friendly (EF), Teaching Friendly (TF), Peer Friendly (PF), and Children Participation (CP). The four factors explained 61.54% of the total variance. Table 3 presents a list of items that were retained and their loading values.

**Table 3:** Exploratory factor analysis for the Child-Friendly School Environment Questionnaire (*N* = 696).

Item	Rotated component matrix				
	F1	F2	F3	F4	Loading
1. I love the dining environment in school.	0.72				0.61
2. Delicious food is served in the school cafeteria.	0.71				0.59
3. There are places in school that I like to read.	0.67				0.53
4. The school is accessible for a small number of physical disabilities or injuries.	0.66				0.53
5. There are professional medical facilities in school.	0.65				0.53
6. I love the music on the school radio.	0.65				0.52
7. A variety of events are often held in school.	0.64				0.55
8. There are plenty of sport areas in school.	0.64				0.43
9. Convenient disinfection facilities in school (e.g., hand sanitizer).	0.62				0.43
10. The teacher will respect my opinion.		0.84			0.84
11. Teachers do not ignore my need.		0.82			0.77
12. The teacher will take the initiative to care about my feelings.		0.81			0.77
13. The atmosphere in our class is united.			0.82		0.78
14. Classmates get along very well.			0.81		0.81
15. Classmates encourage each other.			0.75		0.72
16. I have the right to choose my preferred seat.				0.72	0.59
17. I satisfy with the current position of my seat.				0.71	0.59
18. I could participate in the formulation of class rules.				0.54	0.61
19. I could participate in the design and decoration of the school environment.				0.51	0.50
Number of items	9	3	3	4	
Eigenvalues	7.76	1.66	1.23	1.04	
% of Variance	24.48	13.95	12.42	10.69	
Cumulative % of Variance	24.48	38.43	50.85	61.54	

F1 is Environment Friendly (EF). F2 is Teaching Friendly (TF). F3 is Peer Friendly (PF). F4 is Children Participation (CP).

### 3.3 Analysis of formal test

#### 3.3.1 Model fit assessment

To verify the validity of the four-factor model proposed by the EFA, a confirmatory factor analysis (CFA) was carried out on Sample 3 (*N* = 807) using the 19-item questionnaire. One common fitting optimization index is the chi-square freedom ratio (*χ*^2^/df), which takes into account the complexity of the model and is considered a good fit with an index lower than 4 (Pendergast et al., 2017). However, when the sample is large, the chi-square value may fluctuate due to the sample size, necessitating a significant value of *p* (Jorgensen et al., 2018). Therefore, multiple indicators should be combined to make a comprehensive judgment (Hu and Bentler, 1999; Worthington and Whittaker, 2006; Kline, 2016). Root mean square error of approximation (RMSEA) assesses how well the model fits the population covariance matrix while considering sample size and model complexity. RMSEA values less than 0.06 indicates excellent fit, while values between 0.06 and 0.08 indicate adequate fit (Browne and Cudeck, 1993; Fabrigar et al., 1999). The standardized root means square residual (SRMR) measures discrepancies between covariance matrices of the model. SRMR values less than 0.10 indicate adequate fit, while values below 0.08 indicate good model fit (Hu and Bentler, 1999). Additionally, other fit indices such as the goodness of fit index (GFI), adjusted goodness of fit index (AGFI), incremental fit index (IFI), Tucker-Lewis index (TLI), and comparative fit index (CFI) were considered (Bentler, 1990; Byrne, 2001). Based on these fit indices, the validation of this questionnaire factor analysis supports the results of the exploratory factor analysis that the structure of the child-friendly school environment is reasonable. The values of the above indicators are presented in Table 4.

**Table 4:** Model fit assessment of a confirmatory factor analysis model for a Child-Friendly School Environment Questionnaire.

Fit statistic	Fit recommendations	Test value
Degree of freedom		146
*χ*^2^ /df	< 4	3.92
RMSEA	≤ 0.06	0.06
SRMR	< 0.08	0.05
GFI	> 0.9	0.93
AGFI	> 0.9	0.91
IFI	> 0.9	0.92
TLI	> 0.9	0.90
CFI	> 0.9	0.92

Reliability analysis was utilized to assess the consistency and stability of questionnaire items. The reliability of the questionnaire is primarily determined by calculating Cronbach’s coefficient alpha of the questionnaire. A Cronbach’s alpha above 0.70 is deemed acceptable, above 0.80 is considered good, and above 0.90 is condsidered excellent (Nunnally and Bernstein, 1994; DeVellis, 2016; Thomas and Ganesan, 2020). Reliability analysis was performed on both EFA and CFA (see Table 5).

**Table 5:** Internal consistency reliability coefficient of each Factor in Child-Friendly School Environment Questionnaire.

Factors	EFA Cronbach’s α (*N* = 696)	CFA Cronbach’s α (*N* = 807)
Environment Friendly (EF)	0.82	0.76
Teaching Friendly (TF)	0.88	0.84
Peer Friendly (PF)	0.80	0.83
Children Participation (CP)	0.82	0.84
Total score	0.78	0.87

#### 3.3.2 Confirmatory factor analysis (CFA)

**Figure 1:**
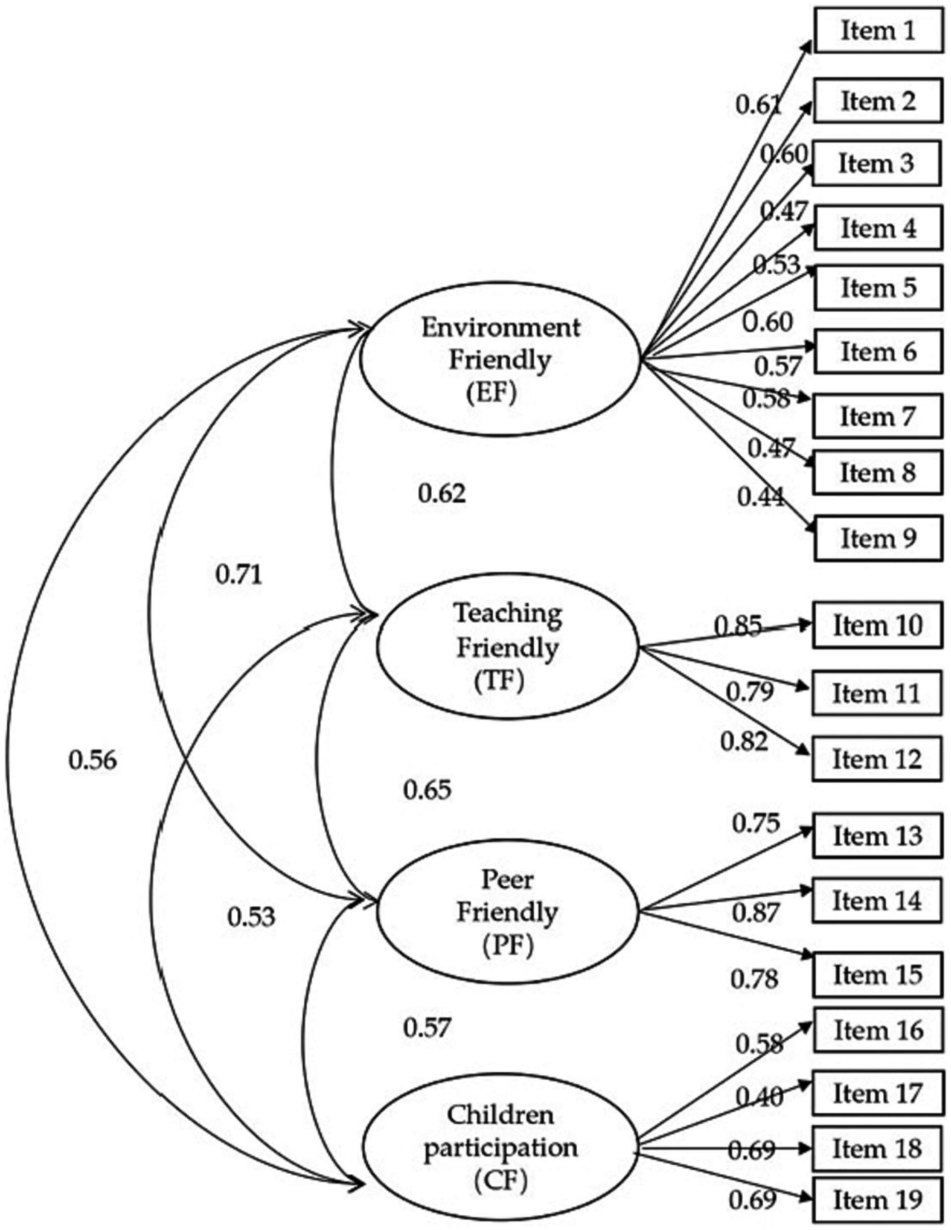
The structural equation model of the Child-Friendly School Environment Questionnaire (*N* = 807).

A confirmatory factor analysis (CFA) was conducted to validate the questionnaire’s structure. The CFSEQ is a questionnaire consisting of four-dimensional constructs. A structural equation model (SEM) was created using the maximum likelihood method (see Figure 1). All items had a factor loading above the standard of 0.4, indicating satisfactory factor validity. The overall fitness of the model was good, making it a suitable evaluation tool for measuring the child-friendliness of the school environment. The structural model includes both measurement and path models. The results suggested that a child-friendly school environment is a multi-dimensional construct including Environment Friendly (EF), Teaching Friendly (TF), Peer Friendly (PF), and Children Participation (CP).

## 4 Discussion

### 4.1 Dimensions of the CFSEQ

The current study developed a 19-item of Child-Friendly School Environment Questionnaire (CFSEQ), which consisted of four factors, that is, Environment Friendly, Teaching Friendly, Peer Friendly, and Children Participation. The results of internal consistency, construct validity, fit indices of the exploratory factor analysis and confirmatory factor analysis have met the requirements of measurement and have good reliability and validity, and the CFSEQ can be used as an evaluation tool for measuring the child-friendliness of school environment.

A child-friendly environment is an educational setting that stimulates children’s natural curiosity and encourages them to take ownership of their own learning path while fostering a sense of responsibility (Shikha, 2021). Child-friendly is society’s comprehensive efforts to prioritize children’s well-being in various aspects, such as hygiene, nutrition, health, education, environment, rights, interests, and participation. This approach is an integrated one, prioritizing children’s well-being across multiple sectors such as health, education, welfare, legal protection, and the built environment. The primary focus is always on the best interests of the child.

The four dimensions of a child-friendly school environment — Environment Friendly, Teaching Friendly, Peer Friendly, and Children Participation — are generally considered to be independent but also interconnected. Each dimension considers the needs and perspectives of children themselves from different angles. Collectively, they contribute to the overall development of a child-centered approach to sustainable development planning.

#### 4.1.1 Environment Friendly

Environment Friendly is a concept that emphasizes the integration of the natural environment with human activities, aiming to create a safe, comfortable, diverse, and interesting environment that considers the needs of all members of the school community. It embodies the centrality of building a child-friendly school environment, including a friendly space for learning and play (Shi and Huang, 2021). This approach not only ensures that the physical environment of the school meets the academic and personal needs of children but also takes into account their emotional and social well-being.

To create a child-friendly school environment, it is essential to listen to the voices of children and incorporate their ideas and suggestions. Children’s perspectives on what constitutes an ideal school environment are valuable and can provide insights into their daily experiences and learning preferences. For example, one way to make learning fun and engaging is to integrate the curriculum with the school’s natural environment. Printing quotes or verses from ancient poetry on campus staircases or walls can help children appreciate the beauty of language while promoting a love for learning. Moreover, providing opportunities for children to participate in various competitions and activities is a great way to engage them emotionally. Announcing public contests on campus can pique children’s interest and make them feel proud of their achievements. Additionally, bringing small animals like sheep, rabbits, etc., onto campus allows children to interact with them during their free time, providing a natural reprieve from their hectic schedules.

Building a child-friendly school environment is not just about creating a conducive space for learning but also about fostering a sense of belonging and community among children, teachers, and other members of the school community. By prioritizing the needs of all stakeholders, schools can create an Environment Friendly space that stimulates children’s interest in learning, enhances their well-being, and prepares them for success in their future endeavors.

#### 4.1.2 Teaching Friendly

Teaching Friendly is an important aspect of promoting a child-friendly learning environment as it focuses on meeting the individual needs of students while fostering their development. It means a respectful, intelligent, and targeting service (Shen et al., 2020). This approach tailors teaching methods to the unique learning styles and interests of each child, leading to more effective and engaging instruction.

In order to establish a Teaching Friendly atmosphere, teachers should exhibit respect towards students, avoid resorting to punishment and scolding, and avoid causing unnecessary delays in the classroom. These measures not only promote a positive classroom atmosphere but also enhance children’s motivation to learn. By creating a supportive teaching environment, teachers can help children feel valued and respected, which is crucial for their development. A Teaching Friendly approach allows teachers to personalize their teaching methods and address the unique needs of each child, thereby promoting their learning and growth.

Moreover, a Teaching Friendly approach goes beyond the classroom setting and extends to the overall school environment. This approach encourages teachers to collaborate with other educators, parents, and school administrators to create a supportive and inclusive learning community. By fostering strong relationships with parents, teachers can gain a better understanding of students’ needs and support their development outside of the classroom.

In conclusion, Teaching Friendly is a critical component of establishing a child-friendly campus environment. By prioritizing the individual needs of students, fostering mutual respect between teachers and students, and adapting teaching methods to maintain students’ psychological engagement, schools can create an environment that encourages learning and promotes students’ success.

#### 4.1.3 Peer Friendly

Peer Friendly is an important concept that emphasizes the importance of addressing children’s needs and perspectives to establish friendly, healthy, and harmonious peer relationships, creating a positive campus atmosphere. As children grow, their peer relationships become increasingly significant, becoming the most critical interpersonal relationship for them.

Friendly peer relationships have a direct impact on the explicit social values and identity of adolescents while providing essential emotional support to help them navigate the challenges of adolescence. In contrast, poor peer relationships, characterized by experiences of rejection and bullying, can have a multitude of detrimental consequences. These include an elevated risk of developing depression and a hindrance to cognitive development, with the potential for these negative effects to endure into adulthood.

Children express their desire to establish positive relationships with their classmates and peers. When faced with adversity and distress among their peers, individuals demonstrate a genuine concern and willingness to provide support. Likewise, when they find someone in need, they readily extend assistance to others.

In conclusion, Peer Friendly is a concept that prioritizes the needs and perspectives of children to establish positive peer relationships and create a campus environment that is friendly, healthy, and harmonious. By promoting friendly peer relationships, schools can help children navigate the challenges of adolescence and develop social skills and emotional support that are essential for their growth and development.

#### 4.1.4 Children Participation

Children Participation is an important concept that emphasizes the importance of listening to children’s voices, considering their needs, and adopting their opinions reasonably in some important school decision-making processes such as planning, evaluating, and policy setting. It is a fundamental right of children, and those who are assertive and capable are encouraged to express their opinions freely. Children’s views should be treated appropriately according to their age and maturity (Jin and Rao, 2022).

Children Participation is multi-situation participation, such as in classroom instruction, in classroom management, and in the improvement of the school environment, etc. In the open-ended interview, the children expressed their desire to have the autonomy to select their own desk partners, actively contribute to the development of the class management system and the enhancement of the campus environment and voice their opinions through the feedback channels provided by the class cadre, class teacher, and administrative staff.

Children’s participation is not only a right but also an important way to promote their development. It can help children develop their communication skills, decision-making ability, and social skills, and improve their self-esteem and self-confidence. Therefore, schools should establish appropriate participation channels and provide support for children’s participation.

### 4.2 Contributions of the current study

The current study has made several contributions. First, the CFSEQ provides a valid and reliable tool for assessing the quality of the school environment from the perspective of children. This instrument can be used by researchers and practitioners to gather information about the school environment and understand children’s experiences and perspectives. Second, the study has shown that the CFSEQ can be used to investigate various factors related to a child-friendly school environment. This approach can help identify areas that need improvement and guide the development of more child-friendly school environments. Finally, the study has demonstrated that the CFSEQ can be used to evaluate the effectiveness of interventions and programs aimed at creating more child-friendly schools, thereby providing valuable feedback and guidance for practitioners and policy makers.

### 4.3 Limitations and the future research

However, it is essential to acknowledge that the current study does have certain limitations. First, the sampling was relatively homogeneous, and the sample size was not large enough to represent the entire country. Future research should consider using larger and more representative samples to increase the generalizability of the findings. Second, the age range of the participants was restricted to students in grades 3 to 6, which limits the applicability of the CFSEQ to younger children or older students. Future research could extend the age range by using visual aids or other methods to accommodate different age groups. Finally, the CFSEQ was developed based on Chinese culture, and its applicability to other cultures and languages remains to be determined. Future research should explore the cross-cultural applicability of the instrument and modify it accordingly to ensure its validity and reliability in different cultural settings.

## 5 Conclusion

A child-friendly school is characterized as a place where it is vital to ensure that all individuals uphold their rights and the rights of others in order to create a positive and supportive school environment, which significantly influences children’s development and growth. The current study presented a validated assessment instrument for evaluating the child-friendly school environment from children’s perspective. The findings from EFA and CFA conducted on the structure of a child-friendly school environment, using the child-reported evaluation method, indicate that the structure comprises four dimensions: Environment Friendly (EF), Teaching Friendly (TF), Peer Friendly (PF), and Children Participation (CP), with good reliability and validity. The development process of the child-friendly school environment evaluation tool focuses on a child-centered perspective of child participation, which reflects the core concept of child-friendly--Children Participation. The utilization of this tool has the potential to serve as a robust assessment mechanism for the future construction of child-friendly schools.

## Data availability statement

The raw data supporting the conclusions of this article will be made available by the authors, without undue reservation.

## Ethics statement

The studies involving humans were approved by the Scientific Research Ethics Committee of Hangzhou Normal University. The studies were conducted in accordance with the local legislation and institutional requirements. Written informed consent for participation in this study was provided by the participants’ legal guardians/next of kin.

## Author contributions

EX: Conceptualization, Formal analysis, Project administration, Supervision, Writing – review & editing. MS: Methodology, Software, Investigation, Validation, Data curation, Writing – original draft preparation. KL: Resources, Data curation, Writing – original draft preparation. XZ: Resources, Writing – review & editing. WJ: Conceptualization, Funding acquisition, Visualization, Writing – review & editing.
