# Supplementary material for: Development and validation of Child-Friendly School Environment Questionnaire from Chinese culture
Source: Front Psychol. 2023 Nov 27;14:1288085. doi: 10.3389/fpsyg.2023.1288085 (PMC10711202; doi:10.3389/fpsyg.2023.1288085)
Supplement: Supplementary file 1 [file Data_Sheet_1.pdf]

The original Child Friendly School Environment Questionnaire (36-item)

Item
1. There are plenty of sports areas in the school.
2. There are professional medical facilities in the school.
3. There are reading places I like in the school.
4. The facilities in the school (e.g., height of sinks and stair rails) do not take my needs into account.
5. The sanitary environment in campus is clean and tidy.
6. There are convenient disinfection facilities in the school (e.g., rinse-free disinfectant hand sanitizer).
7. There are plenty of green plants and small animals in the school.
8. There are a variety of functional classrooms in the school (e.g., music classrooms and art classrooms).
9. I have convenient transportation to and from school every day (e.g., a 15-minute school bus service).
10. Delicious food is served in the school cafeteria.
11. The school has accessibility facilities for the few students with physical disabilities or injuries.
12. A variety of events are often held on campus.
13. There is a unique campus culture in the school (e.g., school motto, school badge, school song and school logo).
14. The security guards in the school are very responsible and make me feel safe.
15. I feel free in the school.
16. I feel very happy in the school.
17. I am treated fairly in the school.
18. I love the music on the school radio.
19. I love the dining environment in the school.
20. I am satisfied with my current seat position.
21. I have the right to speak (e.g., ideas can be expressed through the principal's suggestion box).
22. I have the right of choice (e.g., we can choose my favorite after-school activities).
23. I have the right to vote (e.g., we can vote for class officers).
24. I have the opportunity to participate in the design and decoration of the school environment (e.g., event displays).
25. I have the opportunity to participate in the formulation of class rules.
26. I have the opportunity to present my special skills or work to the class.
27. I have the right to choose which seat I like.
28. The teachers don't ignore my needs.
29. The teachers will respect my opinions.
30. The teachers will take the initiative to care about my feelings.
31. The teachers will start and finish classes on time without delay.
32. The teachers punish students by making them stand in the corner and transcribing their homework.
33. There is fighting, verbal abuse, or bullying among classmates.
34. The classmates encourage each other.
35. The classmates get along very well with each other.
36. The atmosphere in our class is united.

The Child Friendly School Environment Questionnaire (19-item)

Item	Dimension
1. I love the dining environment in the school.<br>2. Delicious food is served in the school cafeteria.<br>3. There are reading places I like in the school.<br>4. The school has accessibility facilities for the few students with physical disabilities or injuries.<br>5. There are professional medical facilities in the school.<br>6. I love the music on the school radio.<br>7. A variety of events are often held on campus.<br>8. There are plenty of sports areas in the school.<br>9. There are convenient disinfection facilities in the school (e.g., rinse-free disinfectant hand sanitizer).	Environment Friendly (EF)
10. The teachers will respect my opinion.<br>11. The teachers don't ignore my needs.<br>12. The teachers will take the initiative to care about my feelings.	Teaching Friendly (TF)
13. The atmosphere in our class is united.<br>14. The classmates get along very well with each other.<br>15. The classmates encourage each other.	Peer Friendly (PF)
16. I have the right to choose which seat I like.<br>17. I am satisfied with the current seat position.<br>18. I have the opportunity to participate in the formulation of class rules.<br>19. I have the opportunity to participate in the design and decoration of the school environment (e.g., event displays).	Children Participation (CP)
